# Fear, misinformation, and pharmaceutical messianism in the promotion of compounded bioidentical hormone therapy

**DOI:** 10.3389/frph.2024.1378644

**Published:** 2024-02-29

**Authors:** Robert P. Kauffman, Eric J. MacLaughlin, Lindsay A. Courtney, David D. Vineyard

**Affiliations:** ^1^ Department of Obstetrics and Gynecology, Texas Tech University Health Sciences Center School of Medicine, Amarillo, TX, United States; ^2^ Department of Pharmacy Practice, Texas Tech University Health Sciences Jerry H. Hodge School of Pharmacy, Amarillo, TX, United States

**Keywords:** bioidentical hormone therapy, compounding, drug safety, menopause, medical populism, pharmaceutical messianism

## Abstract

Compounded bioidentical hormone therapy (cBHT) for menopausal symptoms maintains popularity in western countries despite the availability of hormone products in different formulations and dosages produced by pharmaceutical companies with federal oversight. Akin to many populist therapeutic trends in the history of medicine, cBHT advocates tend to capitalize on consumer fears about existing FDA-approved hormone treatments. Unsubstantiated, exaggerated, or outright false claims are commonplace in promoting cBHT. Given these elements, the basic elements of pharmaceutical messianism continue to drive the cBHT movement.

## Introduction

Historically, the practice of medicine has been riddled with dubious product promotions and ineffective therapies. This phenomenon exists to this day. Contemporary media and online promotions for nutraceuticals (i.e., dietary supplements) for cognitive decline (cleverly sold as memory aids or for brain health), cancer therapies (antioxidants, chelation therapy, and homeopathy), over-the-counter (non-hormonal) testosterone boosters, and anti-aging remedies make unsubstantiated or exaggerated claims to the vulnerable consumer who may be seeking elusive solutions for issues that are part of normal aging or for which current treatments are perceived as unsatisfactory. False or misleading claims and clever advertising jargon skirt federal truth-in-advertising regulations (1–3). Nutraceuticals are regulated as food substances, and while they may not make a health claim such as prevention or treatment of a disease, they make a structure/function claim which may describe the role that the substance may have on the body (4, 5). However, these statements may be misunderstood by the lay public as having data to support prevention or treatment claims. In some cases, patient harm may ensue from drug toxicity or delay in seeking proper care for a potentially serious condition (3, 6).

For menopausal women, black cohosh and other herbals usually fare no better than placebo for treating vasomotor symptoms (VMS), and long-term safety data are lacking (7). In an effort to foster credibility, manufacturers and advertisers promote these products with celebrity spokespersons or real-life users to convey their pitch. Unnamed “top doctors” or “scientific studies” may be touted. These so-called experts usually lack peer-reviewed scholarly accomplishments in the field, and the referenced studies are misinterpreted, poor quality, or even non-existent. Despite unproven benefits, these products account for billions of dollars in annual consumer spending (1, 3, 6).

In the last century, a sociopolitical phenomenon termed “pharmaceutical messianism” has emerged from time to time during health crises or in response to an unanswered critical need in medicine. In such a situation, a drug or treatment protocol is promoted by word-of-mouth, government authority, or social media to solve a health crisis. Promotion of these pharmaceutical panaceas builds on preexisting medical knowledge and practices, borrows from reputable medical authorities (and sometimes heterodoxy), and involves accessible or familiar substances or drugs to treat diseases that do not have an established cure or the risk profile associated with conventional treatments is perceived to be elevated (8). Essentially, pharmaceutical messianism is medical populism where a fashionable solution for a medical disorder is accepted despite limitations in the existing science (9). In some instances, the impetus to adopt pharmaceutical concoctions to control infectious diseases and pandemics contains a political motive (8). Before the development of antiretroviral therapies, African political leaders promoted a host of ineffective nostrums for the treatment of HIV/AIDS for political expediency since no contemporary treatments were effective (8, 9). More recently, the rush to find effective treatments during the worldwide COVID-19 pandemic evolved into a highly politicized effort by world leaders. The promotion of ivermectin and hydroxychloroquine during the COVID-19 pandemic was a *cause celebre* by many physicians, pharmacists, political leaders, and media outlets, thus symbolizing another chapter in the long history of pharmaceutical messianism (8, 10). Cannabis-related products (tetrahydrocannabinol and cannabidiol) have been widely championed to treat innumerable unrelated maladies including psychiatric disorders despite the absence of rigorous clinical trials and safety endpoints (11).

The widespread promotion and utilization of unregulated compounded bioidentical hormone therapy (cBHT) for menopausal symptoms, disease prevention, and longevity is arguably another example of pharmaceutical messianism. The cBHT movement has been accelerated not by a new disease without a cure, but by fear and mistrust of FDA-approved hormone replacement therapy (HRT) and misinformation in the public realm.

## Compounding: an overview

Pharmaceutical compounding maintains a valuable role in population health. Compounding allows the pharmacist to provide medication that might be under a manufacturer shortage, avoid an unwanted component (e.g., gluten or lactulose) or flavor a medication to make it more palatable. The Menopause Society (formerly called the North American Menopause Society), American College of Obstetricians and Gynecologists (ACOG), and other international medical organizations agree that there is a narrow role for pharmaceutical compounding of sex hormones in cases of allergies to US Food and Drug (FDA) approved hormone replacement therapy (HRT) products or the need for a unique dosage or formulation not produced by a government-supervised pharmaceutical company (12, 13). “Bioidentical” has morphed into a marketing term by promoters of cBHT while dismissing the benefits of readily available FDA-approved formulations of estradiol, progesterone, and testosterone with identical molecules in a variety of doses and routes of administration (14, 15).

Before FDA submission and approval, new drugs and drug combinations must be tested for safety and effectiveness. Following animal studies, a novel drug, generic equivalent, or new mode of administration of an established drug must be subjected to a series of clinical trials approved by an Institutional Review Board (IRB) and managed by researchers with expertise in the field. Inclusion and exclusion criteria must be defined, and informed consent obtained from all participants. Data collection must be meticulous, and patient safety must be monitored. An ethical trial design also discloses funding, conflicts of interest, and offers data sharing. Finally, a manuscript is peer-reviewed prior to publication (16, 17). Only then can a New Drug Application (NDA) be made to the FDA.

In the US, state pharmacy boards maintain primary oversight of pharmacies. In 2013, the Compounding Quality Act amended the Food, Drug, and Cosmetic Act in response to the New England Compounding Center tragedy. It allowed the FDA authority to address unlawful or dangerous compounding by companies acting more like manufacturers than state-licensed pharmacies. Under this act, registered outsourcing facilities, which are customarily larger than compounding pharmacies, are exempt from premarket approval requirements if they adhere to current good manufacturing practices, comply with routine FDA inspection, and report adverse events to the FDA. In contrast, traditional compounding pharmacies which produce customized formulations upon receipt of a valid prescription are exempt from these standards (18). There is a concern that both outsourcing and compounding pharmacies circumvent the stricter FDA safety and efficacy oversight to which commercial pharmaceutical companies must comply (19, 20). In a comprehensive 2020 report, the National Academies of Sciences, Engineering, and Medicine (NASEM) urged state pharmacy boards to practice greater oversight of compounding pharmacies (19).

## Fear of conventional HRT

VMS are experienced by 80% of women in various degrees during the menopausal transition with 40% rating them as bothersome and a quality-of-life issue (21, 22). Estrogen is the most effective treatment for VMS with an established safety record in newly menopausal women who do not have a contraindication to systemic therapy (13).

The crisis leading to the meteoric growth of cBHT for menopausal women over the past two decades can be traced to the first publication from the Women's Health Initiative (WHI) in 2002 (23). The British tabloids proclaimed “HRT Attack” and “Millions in HRT Danger” in banner headlines. American media practiced somewhat greater modesty in their journalistic assessment, but the message was the same—a commonly prescribed combination of estrogen and progestogen increased the risk for cardiovascular events and breast cancer, and accordingly, HRT should be considered hazardous (24). Millions worldwide stopped hormone therapy despite established benefits for VMS and genitourinary syndrome of menopause (GSM) (25). Between 2002 and 2020, prescriptions for menopausal hormone therapy dropped 84% in the US without a recovery (26).

The merits and limitations of the WHI study have been extensively debated in the past 22 years since the original preliminary publication. Limitations of the study include, but are not limited to, the selection of the progestogen utilized in the study (medroxyprogesterone), use of a single dose of oral estrogen and progestogen, route of administration (oral), the mean age of the study population, the duration of follow up, dropout rate, and inclusion/exclusion criteria for the study. Scholarly consensus agrees that these elements probably influenced the interpretation of the findings (27). Women enrolled in the first decade after menopause (only a small percentage of the WHI population) benefitted overall in the risk/benefit profile, but this fact received scant attention in the media (13, 24). The finding that conjugated equine estrogens alone might diminish the risk of breast cancer was largely ignored or dismissed as a fluke despite biological plausibility (28–30). Despite benefits in newly menopausal women, public reservations about menopausal HRT persisted. In the new environment of fear and skepticism of conventional hormone therapy, a panacea quickly arose—cBHT.

## Misinformation and pseudoscience

Celebrities, internet communities, and some physicians and pharmacists quickly embraced the purported safety and clinical superiority of cBHT while misrepresenting or misinterpreting the WHI findings and other studies of HRT. Suzanne Somers, an American actress, quickly picked up on the cBHT narrative in a series of books and community lectures. In her book, *Ageless: The Naked Truth About Bioidentical Hormones,* Somers proclaimed that she would never have developed breast cancer if she had utilized cBHT instead of HRT produced by pharmaceutical companies. She offered no credible scientific rationale for this statement. She further stated that cBHT (1) was not a drug, (2) had no harmful consequences, (3) needed no further study, (4) prevented weight gain, (5) possessed anti-aging properties, and (6) prevented multiple conditions such as dyslipidemia, depression, stress, and cognitive decline (31). Her references were largely populist authors with little or no research background or scholarly production. Similarly, many popular books about cBHT written for the consumer rely on unreliable or disreputable sources, incorrect interpretation of the results, and improper extrapolation of the results. There is a notable lack of balance in these publications.

A convenience sample of 100 websites promoting menopausal cBHT in 2017 found numerous claims largely unsupported by the scientific literature. Specifically, cBHT was purported to be less risky compared to identical FDA-approved hormone products, offered anti-aging effects, improved libido, diminished the risk of breast cancer, promoted weight loss, and enhanced fertility (32). The latter claim suggested benefits of cBHT extended to reproductive-aged women. At best, such a claim ignores the fact that progesterone may inhibit the preovulatory luteinizing hormone (LH) surge and that testosterone may promote ambiguous genitalia in the female fetus. Unfortunately, deficiencies in menopause education have created a populist movement ripe with unsubstantiated marketing claims and practices (33). A survey of random websites in 2024 endorsing cBHT found no change in promotion practices from 2017. Perhaps more disturbingly, financial motives associated with cBHT prescribing were common (14, 34).

## cBHT: looking for evidence

In the era of evidence-based medicine, any claim made by cBHT promoters should be established in clinical trials and published in reputable peer-reviewed journals. If, indeed, cBHT is associated with fewer side-effects and better outcomes than conventional hormone therapy, that data should be readily available and open to critical analysis.

It is logical to assume that consumers believe that cBHT products meet similar safety requirements as FDA-approved drugs. The package insert summarizes FDA-approved medications' purity, efficacy, bioavailability, and safety. Non-inferiority and disease treatment and prevention can be drawn from trials published in peer-reviewed journals. In contrast, cBHT products are not required to include prescription labeling with purity, bioavailability, and safety data (19). Despite a recommendation in the NASEM report to add consumer labelling (19), the cBHT industry has largely ignored this proposal. There is a notable absence of quality clinical trials to support non-inferiority, disease prevention, and longevity.

### Purity

Multiple investigators have detected quality issues with compounded hormone products (17, 35, 36). Stanczyk, et al., analyzed compounded creams and capsules from 13 compounding pharmacies and found substantial variations (73%–135%) in the content of estradiol and progesterone among pharmacies. There were even inconsistencies within the same compounding pharmacy (35). Impurities and lack of sterility have been observed in parenterally administered compounded products, sometimes leading to catastrophic outcomes (13, 15, 18, 37).

### Efficacy and bioavailability

In the 2020 NASEM analysis of bioidentical hormone therapy, very little high-quality pharmacokinetic data addressing the absorption, distribution, and metabolism of cBHT creams, pills, and pellets could be found (19).

In a descriptive study of a 50 mg compounded estradiol pellet (*n* = 114) in women with natural and surgical menopause, Wheatley, et al., detected significant variances in serum estradiol levels among the participants with the median return to baseline estradiol level after two implants at 311 days (range 108–1,228 days). In some cases, a single implant produced supraphysiologic estradiol levels for over a year, and abnormal uterine bleeding was a substantial side effect (38). This study illustrates real concerns with the bioavailability of non-FDA approved pellets.

### Safety

The FDA requires new menopausal therapies to demonstrate efficacy against VMS in a 12-week randomized trial and endometrial safety by histological sampling at one year. cBHT formulations avoid such safety oversite. The 2020 NASEM report and 2022 NAMS statement each voice concerns about the substantial dearth of safety data and a lack of randomized trials to prove the safety of cBHT (13, 19).

A retrospective cohort study of 155 menopausal women on FDA-approved hormone therapy and 384 on compounded estradiol pellets (and testosterone pellets in some cases), followed for a mean of 3.5 years, found bothersome side effects or adverse events in 57.6% of the pellet users vs. 14.8% of the FDA-approved product users (*p* < 0.0001). Significantly greater numbers of those using pellets reported anxiety, mastalgia, acne, hair pattern change, weight gain, abnormal uterine bleeding, need for hysterectomy, and mammographic BiRADS score ≥4 compared to FDA-approved products. In addition, mean peak serum estradiol levels were supraphysiologic in the pellet group compared to the FDA-approved hormone arm (237 pg/ml vs. 93.5 pg/ml, *p* < 0.001) (39). Although not a randomized trial, these data are concerning and suggest an urgent need for better safety studies on cBHT.

### Non-inferiority to FDA-approved products

At present, no publications in the peer-reviewed literature demonstrate compounded creams, pills, or pellets to be safer, more efficacious, or less likely to cause adverse events compared to FDA-approved products (12, 13, 19, 40, 41). A properly designed non-inferiority study of a cBHT product vs. a biosimilar FDA-approved product is needed to codify claims made in advertising.

### Disease prevention

Although widely proclaimed in social media and popular books, the multitude of health claims by cBHT producers have not been substantiated by clinical trials and published in reputable peer-reviewed medical journals. Consumer fear of aging, cardiovascular disease, weight gain, Alzheimer's dementia, and breast cancer allows the more simplistic and palatable messages delivered by celebrities and other promoters of cBHT to establish plausible credibility. A positive effect on the length or quality of life, sexual health, and improvement in memory and mood also remains to be established (13, 15, 37, 42).

## Discussion

Science and medicine have evolved into the era of evidence. No convincing evidence exists to validate the belief that cBHT is safer or more effective than HRT, and in fact, available data suggest credible concerns about safety, bioavailability, and purity. Claims made about the favorable effects of cBHT on cardiovascular health, bone loss, VMS, and GSM, are coopted from well-designed studies of FDA-approved HRT that were subject to the stringent FDA approval process and regulatory oversight. To state that these same findings somehow apply equally and automatically to cBHT is hard to reconcile. If cBHT is safer or conveys benefits not associated with conventional HRT, where is the proof? There is ample data to suggest otherwise. If there is a medical contraindication to HRT, the same contraindication would apply to cBHT.

Public fascination with cBHT stems from fear of HRT side effects and the perception that cBHT is the solution to every drawback associated with HRT. Furthermore, prevention of weight gain, breast cancer, cognitive decline, and other conditions of aging augur almost mystical benefits. cBHT is not a panacea as suggested many books and internet sites. We concur with the writings of Lasco, et al., that cBHT fills a perceived void (8, 9), not so much in the treatment of menopausal symptoms, but in the search for longevity and eternal youth. The medical community has a moral and ethical obligation to protect public health and maintain scientific credibility (14). Unless future research demonstrates an unambiguous advantage over conventional HRT, cBHT has earned a place in the annals of pharmaceutical messianism.
